# Study on the Origin and Evolution of Femtosecond Laser-Induced Surface Structures: LIPSS, Quasi-Periodic Grooves, and Aperiodic Micro-Ridges

**DOI:** 10.3390/ma16062184

**Published:** 2023-03-09

**Authors:** Asghar Ali, Piotr Piatkowski, Ali S. Alnaser

**Affiliations:** 1 Department of Physics, American University of Sharjah, Sharjah P.O. Box 26666, United Arab Emirates; 2 Materials Science and Engineering Program, College of Arts and Sciences, American University of Sharjah, Sharjah P.O. Box 26666, United Arab Emirates

**Keywords:** femtosecond laser, polarization, surface structuring, LIPSS, LSFL, HSFL, quasi-periodic grooves, micro-ridges

## Abstract

We investigate the evolution mechanisms of the laser-induced periodic surface structures (LIPSS) and quasi-periodic grooves that are formed on the surface of monocrystalline silicon (mono-Si) when exposed to femtosecond laser radiation of different pulse duration, state of polarization, and fluence. The conditions required for producing LIPSS-free complex micro-ridge patterns are elaborated. The LIPSS evolution mechanism is explained in terms of scattering/interference-based phenomena. To establish the basis for our interpretation, single femtosecond pulses of different pulse durations are irradiated on mono-Si. The absence/appearance of LIPSS rudiments is explained in the context of spectral bandwidth and the associated effects on the intensity of the central wavelength. Shorter fs pulses of a wider bandwidth are employed to induce LIPSS-free micro-ridge patterns. It is demonstrated that the resultant micro-ridge patterns depend on the laser fluence distribution and can be manipulated through laser polarization. The curved morphology of LIPSS rudiments and the evolution mechanism of low- and high-spatial frequency LIPSS, i.e., LSFL and HSFL, are discussed. Finally, it is demonstrated that the consolidated quasi-periodic grooves result from HSFL welding together groups of LSFL. Although our findings are based on fs laser interaction with mono-Si, the results can also be applied to many other materials.

## 1. Introduction

The ultrashort pulse laser processing of metals, semiconductors, and dielectrics is accompanied by the spontaneous appearance of laser-induced surface structures (LISS); this encompasses periodic ripples often known as laser-induced periodic surface structures (LIPSS), quasi-periodic grooves, and aperiodic micro-ridges [1,2,3,4]. LIPSS and quasi-periodic grooves are identifiable from their spatial period (Λ) and preferential orientation, whereas micro-ridges are usually recognized from their typical conic geometry. LIPSS typically have Λ less than the vacuum wavelength (*λ*) of the laser. On the other hand, Λ of the quasi-periodic grooves is usually 2–4 times that of *λ* [3]. Contrary to LIPSS and quasi-periodic grooves, micro-ridges are aperiodic and usually appear as concentric conic sections in the laser-modified spot area [5,6].

These laser-induced structures are very important for producing functional surfaces of technological interest. The LISS have been realized in sub-diffraction limit processing [4], optics and photonics [4,7,8,9], modification of electronic properties and band structure engineering [10], energy conversion and storage [11], photovoltaics [3], sensors and actuators [11,12,13], medical implants and other biological applications [14,15], wettability transition [3,16], cryofouling avoidance [17], micropumps [18], and others. Although LISS are important in the drive for technological advancement, the knowledge of how these structures evolve and how to manipulate them is still limited. From a fundamental and industrial perspective, it is imperative to clearly understand the underlying physical mechanism responsible for LISS evolution and their dependency on laser parameters.

Among the laser-induced surface structures, LIPSS on various materials (metals, semiconductors, and dielectrics) have been investigated the most frequently [19,20,21,22,23,24,25]. Based on Λ relative to *λ*, LIPSS are usually categorized into low-spatial frequency LIPSS (LSFL) and high-spatial-frequency LIPSS (HSFL). LSFL are characterized by a period larger than half of *λ*. On the other hand, HSFL possess Λ of almost half of the LSFL or less than half of *λ*. The orientation of LSFL depends on material properties and the state of polarization (SoP). LSFL have been found to orient perpendicular to the laser polarization direction for most metals and semiconductors. Meanwhile, for large bandgap dielectric materials, LSFL have often been observed to orient parallel to the polarization direction [26].

The relationship between how LIPSS emerge from electromagnetic effects and matter reorganization is highly debatable [26]. Since the discovery of LIPSS in 1965 [27], numerous theories about the LIPSS development mechanisms have been proposed. These theories are mainly based on phenomena such as interference-induced inhomogeneous energy deposition due to rough and inhomogeneous surfaces [20,22,28,29], melt hydrodynamic movement [29] and capillary waves freezing [30], exploding plasma [31], thermally induced surface waves and the generation of transient periodic heating patterns [32], self-organization of surface instabilities [33], nanoplasma formation [34], higher harmonics [35,36,37], excitation of surface plasmon polaritons [4,19], changes in refractive index [38], surface plasmon-laser interference [25,39], etc. The multitude of theories on the subject matter points to the controversial nature of the LIPSS evolution mechanism. Due to the several types of ripples that emerge as a result of the laser–matter interaction [30,32,40,41,42,43], there is no consensus on a single unified explanation of the mechanism that satisfactorily describes the origin and evolution of LIPSS [26,36]. A comprehensive description that can adequately address the emergence and evolution mechanism of LIPSS is still unavailable. 

Quasi-periodic grooves are the second most debated type of structure among laser-induced surface structures. Similar to LIPSS, quasi-periodic grooves also develop on metals, semiconductors, and dielectrics [44,45,46,47,48,49,50,51]. Grooves have often been found to orient perpendicular to LSFL and usually require a higher cumulative fluence (*F*) than LSFL to appear [48,49,50]. 

Similar to LIPSS, the physical mechanism responsible for generating the quasi-periodic grooves is also unclear. However, unlike LIPSS, only a limited number of reports have been dedicated to the evolution of quasi-periodic grooves [51]. Most mechanisms that describe grooves evolution are based on melt hydrodynamic movement [46,52,53], the Marangoni effect and hydrothermal waves [54,55], or on spatial energy redistribution resulting from the interaction of the laser beam with a LIPSS-induced surface wave [44]. 

Despite the efforts undertaken to discuss the origins of the quasi-periodic grooves, there is still controversy in reasoning. For example, the hydrodynamic melt movement is much more pronounced for the first pulse [1] because of the relatively smooth starting surface, whereas grooves usually appear for a larger number of pulses [48,49,50]. Therefore, the hydrodynamic movement cannot be the main reason for the grooves’ evolution. Moreover, if gradients in surface tension were the prime reason for the grooves’ growth, the orientation in one particular direction and grooves strengthening with each incident pulse should not be observable [48,49,50]. Similarly, since Λ of grooves is much larger than that of *λ* [3], therefore, the idea of LIPSS-induced surface waves being responsible for groove generation is far-fetched. 

The micro-ridges are the least discussed amongst the laser-induced surface structures. Similar to LIPSS and quasi-periodic grooves, the micro-ridges also tend to emerge universally irrespective of the type of material [1,2]. However, very few studies are dedicated to micro-ridge analysis. Currently, there are no dedicated investigations on manipulating the micro-ridges to acquire desired surface patterns. 

Micro-ridges appear primarily due to surface modification initiated by the dense laser-induced plasma and melt dynamics [39]. The fluence, *F* distribution influences the micro-ridges that develop around the high *F* center in the laser spot. The material non-uniformity or nanoparticles on the surface can also behave as high *F* centers [56,57]. When the pulse *F* is high enough, explosive boiling and phase explosion are the most significant phenomena determining the ablation level. For lower *F*, the ablation mainly depends on mechanisms such as evaporation and melt expulsion from the center of the laser-modified spot area. The melt expulsion from the center is radial and takes the form of micro-ridges upon re-solidification. Meanwhile, a spattering of the melt also does take place. The melt expulsion from the center decreases the thickness of the melt at the center, and thus the center transforms into a depression [5,56,57,58]. 

Considering the scientific and technological importance of LIPSS, quasi-periodic grooves, and micro-ridges, it is paramount to understand the physical origins of how these structures evolve. So far, no single agreeable mechanism can explain the origins of LIPSS and grooves. Engineering these structures is equally important. We believe that a holistic study entailing a simultaneous investigation of the evolution of the three major LISS structures is required to provide a complete interpretation of the physical mechanisms responsible for LIPSS and grooves evolution. 

This study considers mono-Si as the case study material; however, similar LISS have been observed on other materials as well [2,22,24,44,45,46,47,49,59,60]. Si was selected because of its technological importance [61,62,63,64,65,66] and the availability of rich literature on this material [5,30,50,56]. In this study, we experimentally investigate the physical mechanisms behind the evolution of LIPSS and quasi-periodic grooves. The conditions required for producing LIPSS-free complex micro-ridge patterns are elaborated. LIPSS evolution is explained in terms of an interference/scattered surface wave along the mono-Si surface. We conduct single pulse experiments with pulses of different duration (*τ*), state of polarization, and fluence (*F)*, in order to correlate spectral bandwidth with the appearance of LIPSS. Further, we demonstrate that LIPSS-free laser-modified spot areas can be produced with wider bandwidth and shorter *τ* pulses. It is shown that the modified spot areas can be decorated with LIPSS-free micro-ridge patterns of different distributions by manipulating the SoP of the short fs pulses. Complex micro-ridge patterns distributed in a donut-type space are demonstrated for radial and azimuthal SoPs. To further investigate the evolution of LIPSS and quasi-periodic grooves, the morphological transformations associated with varying pulse count are studied. Finally, an interpretation of grooves’ evolution in terms of evolving LSFL and HSFL is proposed. 

## 2. Experimental

**Figure 1:**
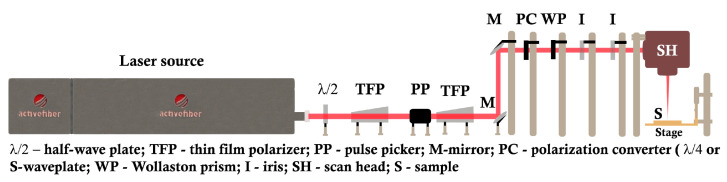
Schematic of the experimental setup.

**Figure 2:**
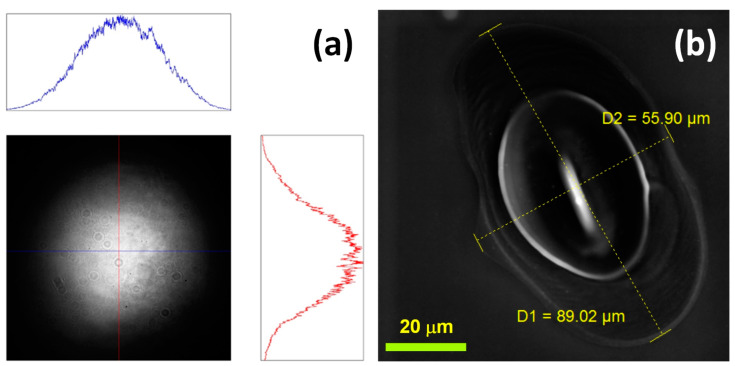
(**a**) Camera captured Gaussian beam profile with plots representing Gaussian distribution profiles along the horizontal and vertical axes (**b**) SEM micrograph of elliptical spot area modified with a single 38 fs pulse of fluence 5 J/cm^2^.

A schematic diagram of the experimental arrangement is shown in Figure 1. A femtosecond laser system (AFSUFFL-300-2000-1030-300, Active Fiber Systems GmbH, Jena, Germany) with a central *λ* of 1030 nm and a repetition rate of 50 kHz was employed. The laser’s output was a linearly polarized Gaussian beam, shown in Figure 2a. The beam power on the sample was controlled by a combination of half-waveplate (*λ*/2) and thin film polarizer (TFP). A pulse picker (MP1-DKDP-11, Eksma Optics, Vilnius, Lithuania), supplied with a timing generator (pMaster 4.2, Eksma Optics, Vilnius, Lithuania) and an external polarizer, were employed to manipulate the laser pulse frequency. The polarization states studied include linear, circular, radial, and azimuthal SoPs. We used appropriate polarization converters (PC), such as quarter waveplate (*λ*/4, Thorlabs Inc., Newton, NJ, USA) and S-waveplate (Altechna, Vilnius Lithuania), to change the pulse polarization from linear to circular, radial, or azimuthal. In the circular SoP, the two orthogonal components from the *λ*/4 plate were spatially separated with a Wollaston prism and equalized by rotating the *λ*/4 plate until equal power was measured for either component. For radial and azimuthal SoPs, the center of the converter was aligned with the optical axis of the incident beam. Moreover, the working axis of the converter was rotated in relation to the incident linear SoP [67]. For radial polarization, the alignment mark on the converter was set parallel to the beam polarization, whereas the azimuthal was aligned perpendicular to the incident beam polarization direction. The beam was focused on the sample by adjusting its position with a *z*-axis adjustable stage. The pulses were spatially isolated with a scan head with adjustable scan speed (v_scan_) and line spacing (Δd).

A wafer of polished p-type mono-Si with 99.65% preferential orientation in the {001} direction was cut into 20 mm × 20 mm size samples for LISS evolution studies. For *F* calculation, the average spot diameter was calculated from the scanning electron micrographs of the spot area modified with 38 fs pulses of the highest *F* (5 J/cm^2^) (Figure 2b) employed in the study. The average diameter (d_spot_) of the ellipse-shaped laser-modified spot area, which equaled 70 µm, was calculated by averaging the major and minor diameters of the ellipse. 

To analyze the effects of τ on the morphological transformation of the single pulse modified spot area, 38 fs and 78 fs pulses were employed. For analyzing the structural transformation of the single-pulse modified spot area at different *F* values, the *F* was varied between 0.13 J/cm^2^ to 5 J/cm^2^. 

The single-pulse SoP-dependent experiments for producing various micro-ridge patterns were performed with 38 fs and 1.56 J/cm^2^ pulses. At *F* close to 1.56 J/cm^2^, well-defined, reproducible micro-ridges patterns could be observed for linear, circular, radial, and azimuthal SoPs. Similarly, the multiple pulse experiments with N from 1 to 200 were carried out with 78 fs pulses of *F* 0.7 J/cm^2^. 

The 2D fast Fourier transforms (FFTs) of the scanning electron micrographs were calculated with SEM analysis software (VegaTC SEM). The morphological analysis was conducted with a SEM (TESCAN VEGA 3 LMU). The height profiles result from the pixel intensity analysis of SEM images using OriginPro along a horizontal line passing through the center of the laser-treated area. Hence, the vertical scale does not reflect the actual height variation.

## 3. Results and Discussion

**Figure 3:**
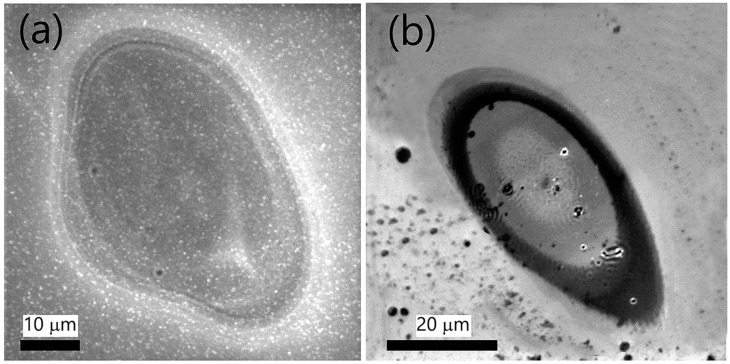
SEM micrographs of spot areas modified with a single linearly polarized femtosecond pulse of fluence 0.7 J/cm^2^ and pulse duration (**a**) 38 fs and (**b**) 78 fs.

To begin, we discuss the results of Si modification by single 38 and 78 fs laser pulses having F 0.7 J/cm^2^ (Figure 3). With a difference of 40 fs, the 78 fs pulse is almost twice as long as the 38 fs pulse. The difference (40 fs) is long enough to analyze the pulse duration-related morphological transformations. Notably, the substrate’s irradiation with a 38 fs pulse (Figure 3a) results in the formation of relatively well-defined micro-ridges, i.e., concentric conic sections occupying the rim of the modified spot area, while there is no signature of LIPSS. On the other hand, LIPSS rudiments can be observed after interaction with a longer pulse (Figure 3b). The results point out that *τ* is a crucial factor in LIPSS evolution by a single femtosecond pulse. 

We believe that LIPSS evolve on ionized media with no translational motion, as long as the media temperature is above the temperature for plasticity transition. In the case of Si, this temperature is 0.6 times its melting point [68]. We can call this phase the cooling melt. As we shall see later, an explanation based on strong hydrodynamic movement due to the high power of the 38 fs pulse, not allowing for LIPSS formation, is not justified. However, the phenomenon can be explained based on the interference patterns resulting from the incident and surface-scattered wave interaction.

The *τ* dependence of surface structures can be explained in terms of surface modification because of interference patterns that result from the interaction of the scattered surface wave and the incident laser radiation [26,69]. It is known that nano-rough features result in the scattering of the incident laser radiation leading to a surface wave that can interfere with the incident radiation [69]. The substrate absorbs the spatially redistributed energy, and if the *τ* and energy of the incident pulse are large enough, then periodic structures are triggered upon relaxation of the substrate [26,69]. In the context of the current study, both spectral bandwidth associated with pulse duration and light dispersion may influence the appearance of periodic patterns on Si. In this regard, it was shown by Rudenko et al. [69] that for shorter fs pulses, pulse duration, which is related to spectral bandwidth, is more important and can significantly affect the interference patterns, and thus the laser-induced surface features. In the current study, we observed that the narrowing of the bandwidth from approximately 47 to 26 nm, associated with a change in *τ* from 38 to 78 fs, resulted in the appearance of LIPSS rudiments. Since shorter pulses are associated with wider bandwidths, shorter pulses have smaller amplitudes around the central wavelength, and thus relatively lower central energy is absorbed by the target surface [69]. Hence, the photoinduced features are less intense and, therefore, more dispersed, due to the contributions from the shifted wavelengths associated with shorter fs pulses. In the case of 38 fs pulses, the LIPSS did not appear, or their amplitude was so small that they could not be identified, whereas the 78 fs pulses were long enough to induce LIPSS rudiments. Since LIPSS evolves in response to the interference patterns influenced by the central *λ*, the spatial period Λ LIPSS is usually comparable to or lesser than *λ*. LSFL are due to the fundamental *λ*, whereas HSFL, which often appears at 90° to LSFL, may be attributed to the harmonic components [37].

**Figure 4:**
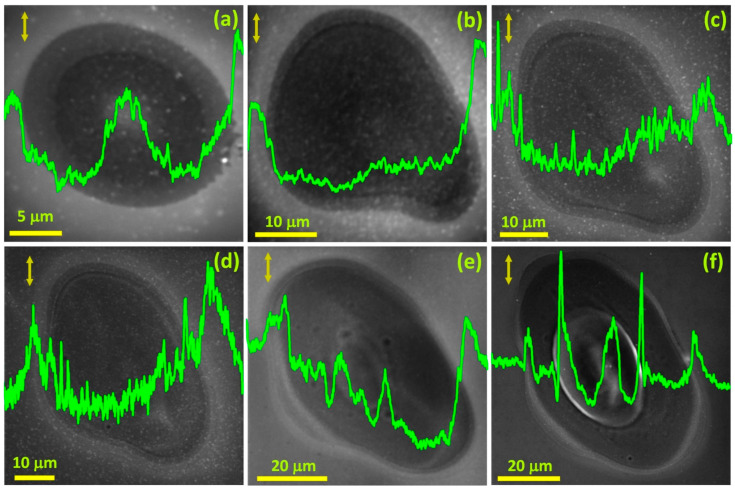
SEM micrographs of spot areas on Si (001) modified with spatially isolated 38 fs linear pulses of fluence (**a**) 0.13 J/cm^2^ (**b**) 0.3 J/cm^2^ (**c**) 0.5 J/cm^2^ (**d**) 0.7 J/cm^2^ (**e**) 1.56 J/cm^2^ (**f**) 3 J/cm^2^. The plot on each spot is representative of the height profile along a horizontal line (single pixels array) through the center of the modified spot area. As the height profiles are the result of an intensity analysis per pixel, the obtained values do not reflect the actual heights.

To verify the deduction that pulse power is not the deciding factor, but it is the pulse duration, *τ*, that determines the possibility of LIPSS appearance, we carried out experiments with 38 fs pulses of varying *F*. Figure 4a–f depicts the spot areas on the Si surface modified with single 38 fs pulses with *F* 0.13 J/cm^2^, 0.3 J/cm^2^, 0.5 J/cm^2^, 0.7 J/cm^2^, 1.56 J/cm^2^, and 3 J/cm^2^, respectively. Interestingly, none of the modified spot areas (Figure 4a–f) depict LIPSS rudiments. Although we do not observe any LIPSS rudiments, we can observe features associated with the changing hydrodynamic movement with the changing *F*. It proves that *τ* is a key factor in deciding LIPSS emergence. Figure 4a–f demonstrates that the modified spot area is getting larger with increasing *F*, which is attributed to the availability of a relatively higher *F* on the periphery, sufficient enough to cause structural modifications. Besides spot size, the crater at the center of the spot and the micro-ridges encircling the crater are becoming more visible with increasing *F* (Figure 4a–f, the height profiles). For lower *F*, the micro-ridges are relatively few and faint (Figure 4a–d); however, as we increase *F*, stronger micro-ridges populate the modified spot area (Figure 4e,f). The micro-ridge formation is more pronounced at 1.56 J/cm^2^ and further high *F* values (Figure 4e,f). While the height profiles do not give the actual height values, they do allow observing the fluence-dependent evolution of morphological features on the Si surface.

From the above observations, we infer that micro-ridges usually appear for single femtosecond pulses that are energetic enough to ignite plasma, but are not too high to cause explosive material removal from the hotter sections of the modified spot area. Micro-ridges will appear if *F* is enough to cause melting and evaporation [58]. In the case of explosive material removal, the ablation is too rapid, and the micro-ridges get covered with the ablation debris even if they form.

**Figure 5:**
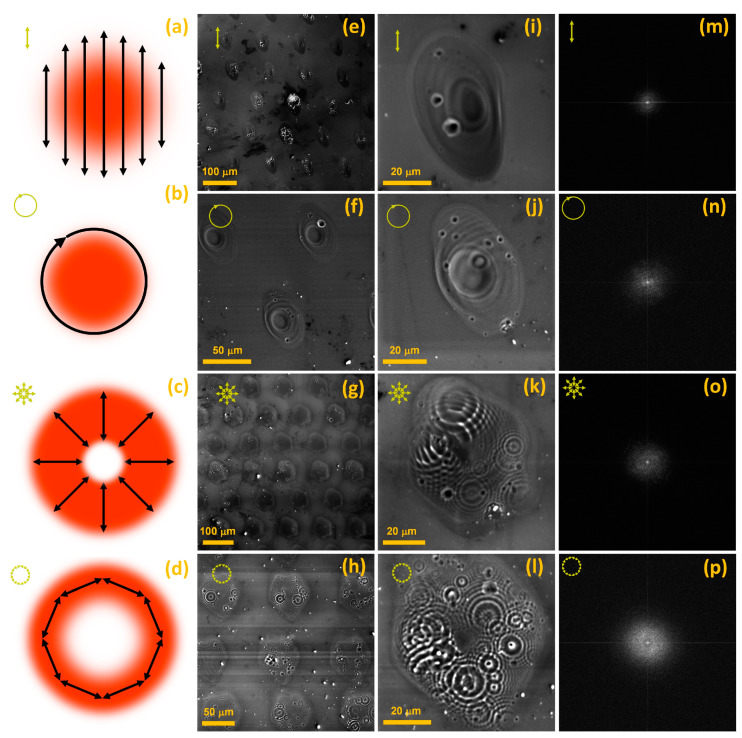
Visualization of the spatial distribution of fluence/electric field of the (**a**) incident linearly polarized Gaussian beam, and its subsequent conversion to (**b**) circularly (**c**) radially and (**d**) azimuthally polarized beams. SEM micrographs of an array of spot areas on Si modified by (**e**) linearly (**f**) circularly (**g**) radially and (**h**) azimuthally polarized single pulses. SEM micrographs of isolated spot area on Si modified by (**i**) linearly (**j**) circularly (**k**) radially and (**l**) azimuthally polarized single pulses. (**m**–**p**) represent the corresponding 2D Fourier transforms of (**i**–**l**).

To further validate our deduction that the 38 fs are inefficient for inducing LIPSS, we changed the SoP (Figure 5a–d). Figure 5e–h presents an array of spatially isolated spot areas modified with isolated 38 fs pulses of *F* 1.56 J/cm^2^ and linear (Figure 5e), circular (Figure 5f), radial (Figure 5g), and azimuthal (Figure 5h) SoPs. Meanwhile, in Figure 5, panel i–l visualizes typical spot areas taken from the respective arrays shown in Figure 5e–h. In Figure 5, panel m–p depicts the corresponding 2D fast Fourier transforms (FFTs) of Figure 5, panel i–l. 

Figure 5a–d details the *F* distribution for the liner (Figure 5a), circular (Figure 5b), radial (Figure 5c), and azimuthal (Figure 5d) SoPs. For the Gaussian beam profile, linear and circular SoPs have maximum *F* occupying the center of the beam (Figure 5a,b), whereas radial and azimuthal SoPs have *F* distributed in a donut space, with minimum *F* at the center of the beam (Figure 5c,d). Such spatial *F* distribution can be achieved by varying the optical retardation with an S-wave plate. The variation in the optical retardation allows one to shift the position and vary the size of the region of minimum *F*, thus bending the direction of the polarization and spatially redistributing *F* [6].

Figure 5e–h points out that the microstructural details of all the laser-modified spots for each SoP are not identical. Although the shot-to-shot power fluctuation of the laser beam might be contributing to it [57], there are other local phenomena, such as material boiling and phase explosion, melting, vaporization, formation of bubbles due to boiling below the surface, oxidation, amorphization, and re-crystallization, that could also contribute [50]. Additionally, the laser-induced nanoparticles also contribute to this spot-to-spot variation [1]. No signature of LIPSS rudiments could be observed. Nonetheless, there is a typical micro-ridge pattern associated with each SoP.

Figure 5i,j describes individual spot areas modified with linear and circular SoPs. By analyzing Figure 5i,j, we observe that linear and circular SoPs produce similar modified spot areas. In either case, the spot center has a crater and is surrounded by micro-ridges that extend radially from the central crater zone. Therefore, it is conceived that, although circular polarizers change the state of polarization, they have minimal effects on *F* distribution. Figure 5k,l depicts individual spot areas modified with radial and azimuthal SoPs, respectively. By examining Figure 5k,l, we find that both radial and azimuthal SoPs produce similar modified spot areas. There is no crater zone in the center, and the micro-ridges are more uniformly distributed throughout the modified spot area. The micro-ridges, in either case, are concentric circles occupying most of the spot area, excluding the central zone. A complex pattern evolves, comprising flower-like partially superimposing concentric micro-ridges distributed along a donut-type space. 

Figure 5m–p presents the 2D Fourier transforms (FFTs) of the laser-modified spot areas shown in Figure 5i–l, respectively. None of the FFTs show any frequencies associated with LSFL or HSFL. There is no fundamental period, Λ, but a range in spatial periods is scattered in all directions. Among the four FFTs (Figure 5m–p), azimuthal SoP demonstrates the highest frequency components. It proves that these micro-ridge patterns are LIPSS-free and aperiodic. 

Although spectral broadening/narrowing-dependent interference/scattering is responsible for LIPSS appearance, how LIPSS orientation changes with the material is yet to be clarified. LIPSS orientation and Λ are material dependent [21]. LSFL usually orient perpendicular to the laser polarization direction on metal surfaces. It appears that materials, such as polymers [20], and large band gap dielectrics, such as diamond [22], BaF_2_ [70], etc., are more inclined towards LSFL, oriented parallel to the direction of laser polarization. However, this is not always the case. For instance, c-Al_2_O_3_ (band gap ~ 9.9 eV) depicted perpendicularly oriented LSFL relative to the laser polarization direction [60]. Some studies showed that a linearly polarized beam produces LSFL with different periodicity directions even for the same material. An example is a-SiO_2_, where LSFL, parallel [70] and perpendicular [59] to the laser polarization, have been reported. 

From the discussion above, we conclude that, besides the processing parameters i.e., *λ*, *τ*, and *F*, material properties are also crucial to LIPSS evolution. The nature of the surface scattered wave appears dependent on material properties, especially its electrical and optical properties. LIPSS orientation depends on the surface reconstruction that results from the interference patterns induced by the incident radiation and the resultant scattered wave. 

**Figure 6:**
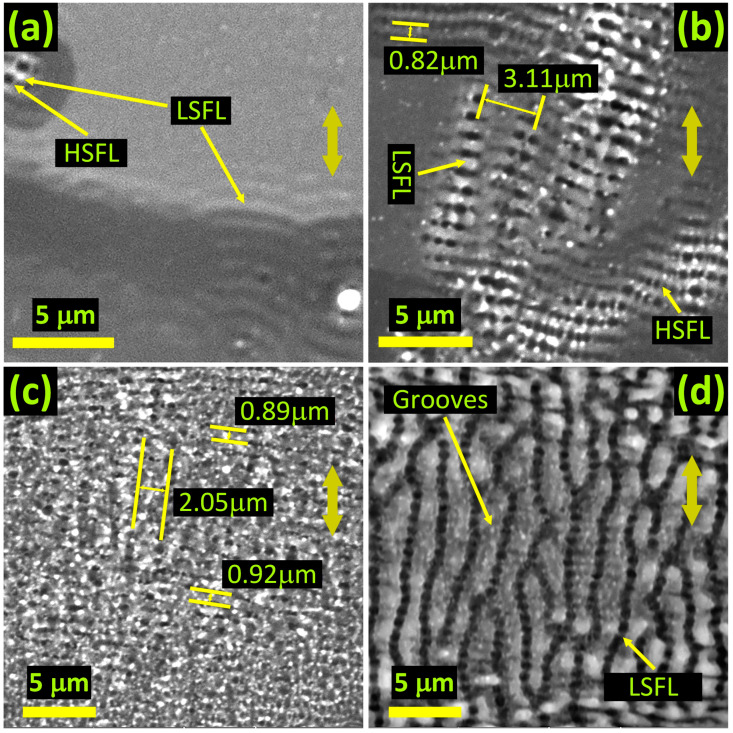
(**a**) SEM micrographs of laser-modified spot areas irradiated with (**a**) N = 5, (**b**) N = 10, (**c**) N = 20, and (**d**) N = 200 pulses of 78 fs and 0.7 J/cm^2^ fluence.

Figure 6 shows selected images of spot areas modified with 78 fs linear pulses of 0.7 J/cm^2^. Figure 6a depicts the presence of both LSFL and HSFL in the spot area modified with 5 pulses. The LSFL and HSFL, wherever they exist together, appear to giving rise to the grooves’ rudiments. In addition, LSFL are curvy at this early stage of development. Figure 6b depicts the spot after 10 pulses and shows evolving grooves at sites where LSFL and HSFL coexist. After 20 pulses, as shown in Figure 6c, the LSFL, HSFL, and grooves seem to coexist but are welded together. For the 200 shots modified spot area shown in Figure 6d, well-developed quasi-periodic grooves and LSFL coexist, with no signature of HSFL. The LSFL are aligned perpendicular, whereas the grooves are aligned parallel to the laser polarization direction.

We believe that LIPSS rudiments are curved (Figure 6a) primarily because of concentric interference patterns that emerge around nano-irregularities [69]. In addition, the cooling melt pool exhibits uniform properties at fixed radial distances from the high *F* center. The uniform themofluic and thermomechanical properties at constant radial distances facilitate such curvatures.

As shown in Figure 6a, HSFL accompany LSFL right from the first few pulses. However, HSFL are relatively weak and have a characteristic Λ even shorter than that of LSFL (Figure 6b). We observe that the quasi-periodic grooves emerge due to the strengthening of HSFL with N (Figure 6b,c). HSFL weld together groups of adjacent LSFL to give rise to consolidated groove structures. Since surface reconstruction does not favor smooth surfaces, grooves beak at Λ, which is usually 2–4 times that of *λ* (Figure 6d).

## 4. Conclusions

We have investigated the morphological evolution of LIPSS, quasi-periodic grooves, and micro-ridges induced on Si surface by femtosecond laser pulses. A physical interpretation of the structural transformations as a function of pulse duration, pulse fluence, number of pulses, and state of polarization, was presented. It was shown that the duration of the laser pulse had a pronounced effect on the appearance of LIPSS. For shorter femtosecond pulses (38 fs), no LIPSS rudiments were observed. However, for relatively longer pulses (78 fs), LIPSS rudiments appeared right from the first pulse. In the case of multiple pulse laser-Si interactions, it was found that both LSFL and HSFL were enhanced with each subsequent pulse. Similarly, quasi-periodic grooves were found to emerge due to HSFL welding together groups of neighboring LSFL, whereas micro-ridges were found to depend on the pulse fluence.

Moreover, it was deduced that the LIPSS spatial period is influenced by the energy deposition patterns that result from the interference of the incident radiation and the scattered wave. Due to spectral broadening and dispersed interference patterns associated with the shorter pulse (38 fs), LIPSS were not induced. Similarly, the orientation of LIPSS was thought to depend on the materials’ electrical and optical properties. Likewise, LSFL and HSFL were correlated with the central wavelength and harmonic components, respectively. LIPSS-free complex micro-ridge patterns, distributed in a donut-type space, were induced with shorter pulses of radial and azimuthal polarizations. The fluence distribution in the laser-modified spot area determined the micro-ridge patterns created in the modified spot area. The insights provided here are of paramount importance in realizing controlled and reproducible surface features of technological significance.

## Data Availability

Any further details relevant to this study may be obtained from the authors upon a reasonable request.
